# Supplementary material for: Safety and immunogenicity of a subtype C ALVAC-HIV (vCP2438) vaccine prime plus bivalent subtype C gp120 vaccine boost adjuvanted with MF59 or alum in healthy adults without HIV (HVTN 107): A phase 1/2a randomized trial
Source: PLoS Med. 2024 Mar 19;21(3):e1004360. doi: 10.1371/journal.pmed.1004360 (PMC10986991; doi:10.1371/journal.pmed.1004360)
Supplement: S1 Text — (PDF) [file pmed.1004360.s002.pdf]

S1 Text: Supplemental methods

Intracellular cytokine staining (ICS) to measure Env-specific CD4+ T-cell response

Peripheral blood mononuclear cells, collected at month 6.5, 12, 12.5 and 18 timepoint were isolated and cryopreserved from whole blood as previously described (1). T cell responses to the vaccine matched Clade C Env proteins ZM96 gp140, 1086 gp120, TV1 gp120 and Gag-LAI were measured using the intracellular cytokine staining assay as previously described (2,3), using a 17-color ICS panel (4). Briefly, cryopreserved PBMC were thawed, incubated overnight and stimulated on day 2 for six hours at 37°C with either peptide pools (peptides of 15 amino acids overlapping in sequence by 11 amino acids), dimethyl sulfoxide (DMSO, 0.5%, Sigma Aldrich; negative control) or staphylococcal enterotoxin B (SEB, 0.25 µg/mL; Sigma Aldrich; positive control) in the presence of costimulatory antibodies CD28 and CD49d (1 µg/ml, BD Biosciences) and brefeldin A (BFA, 10 µg/ml, Sigma Aldrich). Cells were incubated with ethylenediaminetetraacetic acid (EDTA, 2 mM, Life Technologies) overnight at 4°C, then stained with a 17-color antibody staining panel and acquired on a BD Fortessa flow cytometer (BD Biosciences) and data were analyzed using FlowJo version 9.9.4 (FlowJo LLC).

	Specificity	Fluorochrome	Clone	Manufacturer	Catalogue number
Viabilty marker	AViD	NA	NA	Life Technologies	L34957
Cellular surface markers	CCR7	BV785	G043H7	BioLegend	353229
	CD14	BV510*	M5E2	BioLegend	301842
	CD56	BV570	HCD56	BioLegend	318330
	CD45RA	APC H7	HI100	BD Biosciences	560674
	CXCR5	PE-Dazzle594	J252D4	BioLegend	356928
	ICOS (CD278)	BV711	DX29	BD Biosciences	563833
	PD-1 (CD279)	BV605	EH12.2H7	BioLegend	329924
Intracellular markers	CD3	BUV737	UCHT1	BD Biosciences	564307
	CD4	BUV395	SK3	BD Biosciences	563550
	CD8	BV650	RPA-T8	BD Biosciences	563821
	CD154	APC	TRAP-1	BD Biosciences	555702
	IFNγ	V450	B27	BD Biosciences	560371
	Granzyme B	Alx700	GB11	BD Biosciences	560213
	IL-2	PE	MQ1-17H12	BD Biosciences	559334
	IL-4	PerCP-Cy5.5	MP4-25D2	BioLegend	500822
	IL-17a	PE-Cy7	BL168	BioLegend	512315
	TNFα	FITC	MAb11	eBioscience	11-7349-82

For a given antigen, marker, and T-cell subset, the “magnitude” of the immune response is measured by the log10 net percent of antigen-specific cells in the T cell subset that express the

marker, comparing antigen-stimulated cells vs. unstimulated cells. Net percentages less than 0.01 are set equal to 0.01 (1/10,000). The following data were excluded from analysis: unreliable samples, visits outside allowable visit window, or the mean negative control responses for IFN-γ and/or IL-2 ≥ 0.1%. Records were excluded if the number of CD4+ T-cell subsets was <10,000 or the number of CD8+ T-cell subsets was <5,000. These criteria were applied separately to CD4+ and CD8+ subsets.

The magnitude of response reported, or "net response," is the difference between the stimulated and the average of the two unstimulated wells of the percent of CD4+ T cells that express at least one of the markers in the subset: IL-2 or IFN-γ or CD40L. This percent was calculated as the sum of the cell counts across all Boolean combinations of the markers divided by the total number of CD4+ T cells. The average of the two unstimulated wells was calculated by summing the percent of CD4+ T cells that express at least one of the markers in the subset: IL-2 or IFN-γ or CD40L and dividing by two.

Binary response variables (i.e., "positive"/ "negative" responses) were defined as follows. To assess positivity for a peptide pool within a T-cell subset, a two-by-two contingency table was constructed comparing the HIV-1 peptide stimulated and negative control data. The four entries in each table are the number of cells positive for IFN-γ or IL-2 or CD40L and the number of cells negative for IFN-γ or IL-2 or CD40L, for both the stimulated and the negative control data. If both negative control replicates are included, then the average of the number of total cells and the average of the number of positive cells was used. A one-sided Fisher's exact test was applied to the table, testing whether the number of cytokine-producing cells for the stimulated data is equal to that for the negative control data. Since multiple individual tests (for each peptide pool) were conducted simultaneously, a multiplicity adjustment was made to the individual peptide pool p-values using the Bonferroni-Holm adjustment method. If the adjusted p-value for a peptide pool was ≤ 0.00001, the response to the peptide pool for the T-cell subset was considered positive. Because the sample sizes (i.e., total cell counts for the T-cell subset) was large, e.g., as high as 100,000 cells, the Fisher's exact test has high power to reject the null hypothesis for very small differences. Therefore, the adjusted p-value significance threshold was chosen stringently (≤ 0.00001).

For the non-overlapping Env ZM96-1 and Env ZM96-2 peptide pools, the magnitude for Env ZM96 gp140 was calculated as the sum of Env ZM96-1 and Env ZM96-2. Both pools were included regardless of positive or negative responder status and magnitudes less than zero were not truncated at zero prior to summation. The overall magnitude was calculated as the sum of the individual Env ZM96-1 and Env ZM96-2 magnitudes and the overall ZM96 response was defined as positive if either Env ZM96-1 or Env ZM96-2 responses were positive.

The COMPASS polyfunctionality score is defined as the estimated proportion of antigen-specific cell subsets detected, weighted by their degree of functionality (5). "Detected" antigen-specific cell subsets are those with a statistically significant difference in the percent of marker-expressing cells in unstimulated vs. stimulated samples. The following markers were used to calculate the polyfunctionality score (PFS): IFN-γ, IL-2, TNF, CD40L, and IL17. For the COMPASS analysis, cell subsets that did not have at least 10 cells in at least 5 participants were excluded. The standard ICS filter on mean negative control is not used. Post-infection samples from participants diagnosed with HIV were excluded after COMPASS filtering. The polyfunctionality score for Env ZM96 gp140 was calculated as the maximum of the Env ZM96-1 and Env ZM96-2 polyfunctionality scores.

Binding antibody multiplex assay (BAMA) to measure binding antibody (bAb) response

The gp120 and V1V2 antigens assessed with BAMA are included in S1 Table. The readout was background-subtracted mean fluorescence intensity (MFI), where background accounts for both an antigen-specific plate level control (i.e., a blank well containing antigen-coated beads run on each plate), and a specimen-specific control (i.e., a serum well containing blank beads). The positive controls were purified polyclonal IgG from HIV-positive subjects (HIVIG) using a 10-point standard curve (4PL fit) and CH58 mAb titration. The negative controls were NHS (HIV-1 seronegative human sera) and blank beads. The sample was repeated if the blank bead negative control exceeded 5000 MFI. If the repeat value exceeded 5000 MFI, the sample was excluded from analysis due to high background. The MFI minus Blank bead responses ("net MFI") at the specified dilutions are used to summarize the magnitude. Net MFI less than 1 was set to 1.

Samples were declared positive if the following held: (1) net MFI ≥ antigen-specific positive response threshold (defined separately for each trial as the maximum of 100 and the 95th percentile of pre-vaccination net MFI values), (2) net MFI > 3 times baseline net MFI, and (3) MFI > 3 times baseline MFI. Net MFI were truncated at 22,000, the upper limit of the linear range of the instrument at a single dilution. Additionally, samples were excluded from threshold calculation and analysis if the baseline analyte net MFI exceeded 6,500.

Statistical analysis

The sample size of the trial provided at least 80% power to address the primary safety and immunogenicity objectives. For example, there is over 80% power to detect a 35% difference in the response rates between the groups primed with ALVAC followed by ALVAC + Bivalent Subtype C gp120 and adjuvanted either with MF59 or with alum by a Fisher's exact two-sided test, with n = 30 per group, assuming a 15% rate of missing data.

Formal statistical comparisons of magnitudes among positive responders were not performed for vaccine groups where the median magnitude at a single dilution was equal to 22,000, the upper limit of the linear range of the instrument.

Data sharing

Data and protocols for HVTN 107 will be made publicly available online at https://atlas.scharp.org/cpas/project/HVTN%20Public%20Data/begin.view.

REFERENCES

1. Marta Bull, Deborah Lee, Jason Stucky, Ya-Lin Chiu, et al. Defining blood processing parameters for optimal detection of cryopreserved antigen-specific responses for HIV vaccine trials. J Immunol Methods. 2007 Apr 30;322(1-2):57-69. doi: 10.1016/j.jim.2007.02.003.

2. Stephen C De Rosa, Donald K Carter, M Juliana McElrath. OMIP-014: validated multifunctional characterization of antigen-specific human T cells by intracellular cytokine staining. Cytometry A. 2012 Dec;81(12):1019-21. doi: 10.1002/cyto.a.22218.

3. Helen Horton, Evan P Thomas, Jason A Stucky, Ian Frank, et al. Optimization and validation of an 8-color intracellular cytokine staining (ICS) assay to quantify antigen-specific T cells induced by vaccination. J Immunol Methods. 2007 May 31;323(1):39-54. doi: 10.1016/j.jim.2007.03.002. Epub 2007 Apr 3.

4. Mina C Hosseinipour, Craig Innes, Sarita Naidoo, Philipp Mann, et al. Phase 1 Human Immunodeficiency Virus (HIV) Vaccine Trial to Evaluate the Safety and Immunogenicity of HIV Subtype C DNA and MF59-Adjuvanted Subtype C Envelope Protein. Clin Infect Dis. 2021 Jan 23;72(1):50-60. doi: 10.1093/cid/ciz1239.

5. Lin L, Finak G, Ushey K, Seshadri C, Hawn TR, Frahm N, et al. COMPASS identifies T-cell subsets correlated with clinical outcomes. Nat Biotechnol. 2015;33(6):610-6.
