# Supplementary material for: Safety and immunogenicity of a subtype C ALVAC-HIV (vCP2438) vaccine prime plus bivalent subtype C gp120 vaccine boost adjuvanted with MF59 or alum in healthy adults without HIV (HVTN 107): A phase 1/2a randomized trial
Source: PLoS Med. 2024 Mar 19;21(3):e1004360. doi: 10.1371/journal.pmed.1004360 (PMC10986991; doi:10.1371/journal.pmed.1004360)
Supplement: S1 Table — (PDF) [file pmed.1004360.s003.pdf]

Table S1: Details of the BAMA and ICS antigens including HIV-1 viral strain information

Assay	Antigen class	Full antigen name	Antigen label used in plot	Viral strain information: Subtype.Country.Year.Stage *
BAMA bAb	gp120	1086C_D7gp120.avi/293F	1086 gp120	C.MW.04.1-2
		TV1c8_D11gp120.avi/293F	TV1 gp120	C.ZA.98.6
		96ZM651.D11gp120.avi	ZM96 gp120	C.ZM.96.6
	V1V2	C.1086_V1_V2 Tags	1086 V1V2	C.MW.04.1-2
		gp70-TV1.GSKvacV1V2/293F	TV1 V1V2	C.ZA.98.6
		gp70-96ZM651.02 V1v2	ZM96 V1V2	C.ZM.96.6
		gp70_B.CaseA_V1_V2	B.CaseA V1V2	B.US.88.6
		AE.A244 V1V2 Tags/293F	A244 V1V2	CRF01_AE.TH.90.6
ICS	gp120	1086 gp120	1086 gp120	-
	gp120	TV1 gp120	TV1 gp120	-
	gp140	ZM96 gp140	ZM96 gp140	-

*Subtype is denoted by a capital letter; country of origin is denoted by the 2 digit International Organization for Standardization code; year isolated is denoted by 2 digits; and stage is denoted by “a” (acute, if Fiebig stage is unknown) or “1”, “2”, “3”, “4”, “5”, or “6” (acute or early chronic, where the number or range corresponds to the Fiebig stage or range of stages when known).
