# Supplementary material for: Safety and immunogenicity of a subtype C ALVAC-HIV (vCP2438) vaccine prime plus bivalent subtype C gp120 vaccine boost adjuvanted with MF59 or alum in healthy adults without HIV (HVTN 107): A phase 1/2a randomized trial
Source: PLoS Med. 2024 Mar 19;21(3):e1004360. doi: 10.1371/journal.pmed.1004360 (PMC10986991; doi:10.1371/journal.pmed.1004360)
Supplement: S2 Table — (PDF) [file pmed.1004360.s004.pdf]

Table S2. CD4+ T-cell responses (measured by expression of IFN-γ and/or IL-2 and/or CD40L) to Gag at months 6.5, 12, 12.5, 18.

	Response Rate	95% CI
**Month 6.5**		
MF59	1/27 (3.7%)	(0.7%, 18.3%)
Alum	0/27 (0.0%)	(0.0%, 12.5%)
Co-admin	1/28 (3.6%)	(0.6%, 17.7%)
None	2/17 (11.8%)	(3.3%, 34.3%)
**Month 12**		
MF59	1/26 (3.8%)	(0.7%, 18.9%)
Alum	2/29 (6.9%)	(1.9%, 22.0%)
Co-admin	1/28 (3.6%)	(0.6%, 17.7%)
None	1/17 (5.9%)	(1.1%, 27.0%)
**Month 12.5**		
MF59	1/27 (3.7%)	(0.7%, 18.3%)
Alum	2/28 (7.1%)	(2.0%, 22.7%)
Co-admin	1/28 (3.6%)	(0.6%, 17.7%)
None	1/16 (6.2%)	(1.1%, 28.3%)
**Month 18**		
MF59	1/24 (4.2%)	(0.7%, 20.2%)
Alum	2/28 (7.1%)	(2.0%, 22.7%)
Co-admin	1/29 (3.4%)	(0.6%, 17.2%)
None	0/15 (0.0%)	(0.0%, 20.4%)
