# Supplementary material for: Safety and immunogenicity of a subtype C ALVAC-HIV (vCP2438) vaccine prime plus bivalent subtype C gp120 vaccine boost adjuvanted with MF59 or alum in healthy adults without HIV (HVTN 107): A phase 1/2a randomized trial
Source: PLoS Med. 2024 Mar 19;21(3):e1004360. doi: 10.1371/journal.pmed.1004360 (PMC10986991; doi:10.1371/journal.pmed.1004360)
Supplement: S3 Table — (PDF) [file pmed.1004360.s005.pdf]

Table S3. CD8+ T-cell responses (measured by expression of IFN-γ and/or IL-2 and/or CD40L) to TV1 gp120 at months 6.5, 12, 12.5, 18.

	Response Rate	95% CI
**Month 6.5**		
MF59	1/27 (3.7%)	(0.7%, 18.3%)
Alum	0/27 (0.0%)	(0.0%, 12.5%)
Co-admin	0/28 (0.0%)	(0.0%, 12.1%)
None	0/17 (0.0%)	(0.0%, 18.4%)
**Month 12**		
MF59	2/26 (7.7%)	(2.1%, 24.1%)
Alum	0/29 (0.0%)	(0.0%, 11.7%)
Co-admin	0/28 (0.0%)	(0.0%, 12.1%)
None	2/17 (11.8%)	(3.3%, 34.3%)
**Month 12.5**		
MF59	2/27 (7.4%)	(2.1%, 23.4%)
Alum	0/28 (0.0%)	(0.0%, 12.1%)
Co-admin	1/28 (3.6%)	(0.6%, 17.7%)
None	2/16 (12.5%)	(3.5%, 36.0%)
**Month 18**		
MF59	0/24 (0.0%)	(0.0%, 13.8%)
Alum	0/28 (0.0%)	(0.0%, 12.1%)
Co-admin	0/29 (0.0%)	(0.0%, 11.7%)
None	0/15 (0.0%)	(0.0%, 20.4%)
