# Supplementary material for: Safety and immunogenicity of a subtype C ALVAC-HIV (vCP2438) vaccine prime plus bivalent subtype C gp120 vaccine boost adjuvanted with MF59 or alum in healthy adults without HIV (HVTN 107): A phase 1/2a randomized trial
Source: PLoS Med. 2024 Mar 19;21(3):e1004360. doi: 10.1371/journal.pmed.1004360 (PMC10986991; doi:10.1371/journal.pmed.1004360)

Figure S1: **Frequency of maximum severity of systemic (A) and local (B) reactogenicity symptoms by group in the safety cohort.** T1: ALVAC vCP2438 at months 0, 1, 3, and 6 with gp120/MF59 at months 3 and 6; T2: ALVAC vCP2438 at months 0, 1, 3, and 6 with gp120/alum at months 3 and 6; T3: ALVAC vCP2438 with gp120/MF59 at months 0, 1, 3, and 6; T4: ALVAC vCP2438 with gp120 at months 0, 1, 3, 6. Grading per DAIDS Table for Grading the Severity of Adult and Pediatric Adverse Events, Version 2.1, July 2017. DAIDS, Division of AIDS.

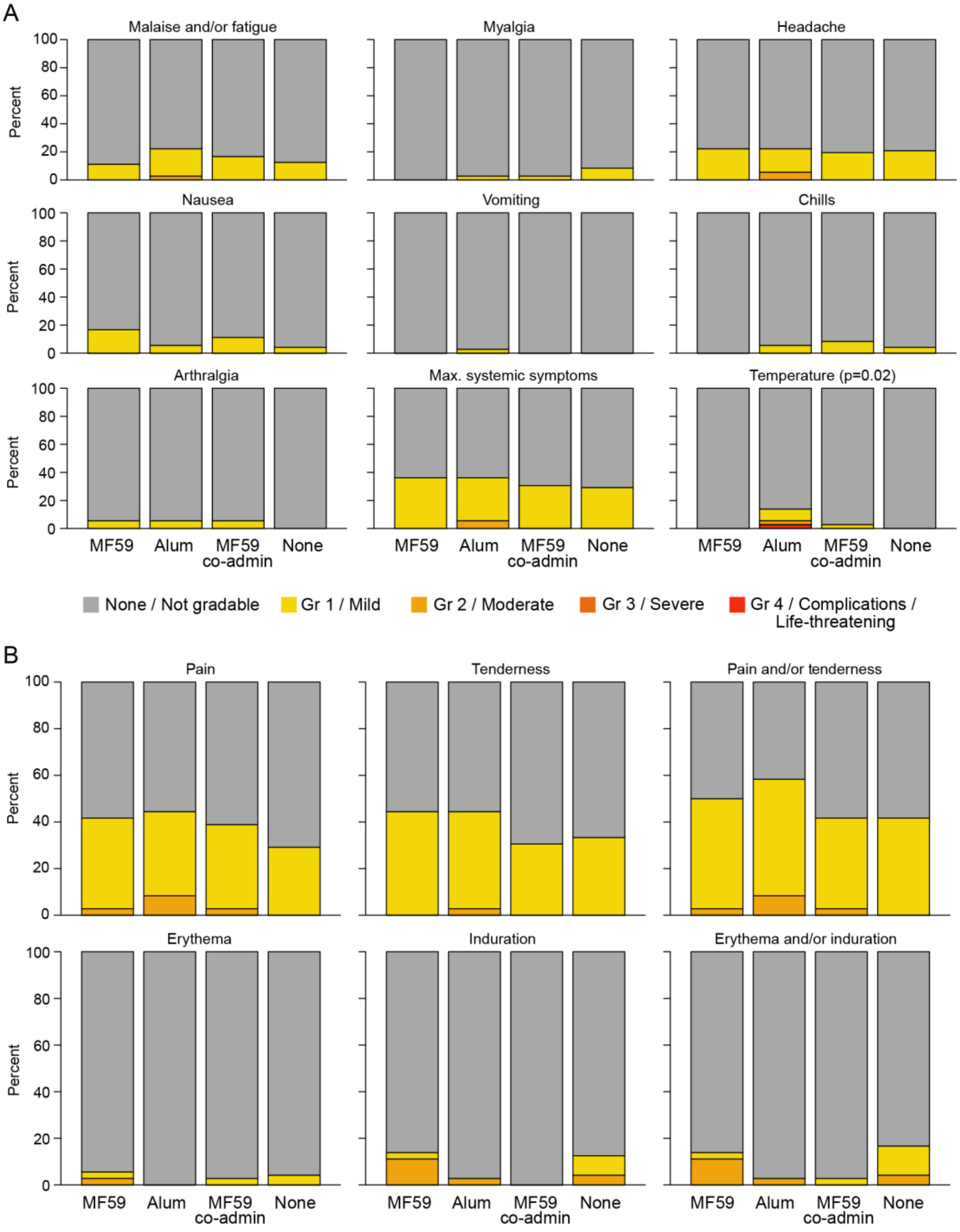

Supplement: S1 Fig — (PDF) [file pmed.1004360.s006.pdf]
