# Supplementary material for: Safety and immunogenicity of a subtype C ALVAC-HIV (vCP2438) vaccine prime plus bivalent subtype C gp120 vaccine boost adjuvanted with MF59 or alum in healthy adults without HIV (HVTN 107): A phase 1/2a randomized trial
Source: PLoS Med. 2024 Mar 19;21(3):e1004360. doi: 10.1371/journal.pmed.1004360 (PMC10986991; doi:10.1371/journal.pmed.1004360)

Figure S2. Serum IgG binding antibody geometric mean titers (AUC) of all participants in all groups at Month 6.5. A. 1086 gp120. B. TV1 gp120.

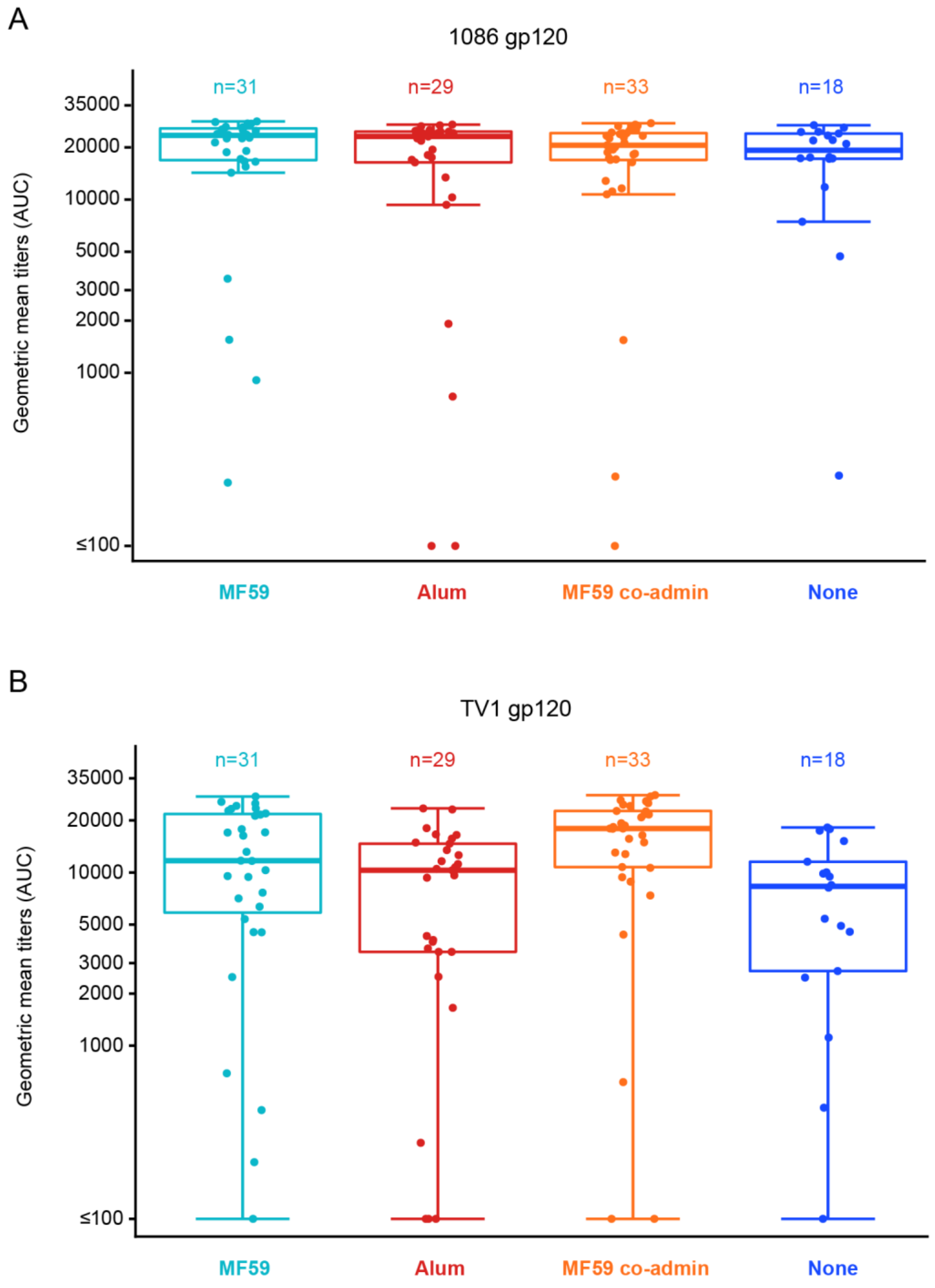

Supplement: S2 Fig — (PDF) [file pmed.1004360.s007.pdf]
