# Supplementary material for: Safety and immunogenicity of a subtype C ALVAC-HIV (vCP2438) vaccine prime plus bivalent subtype C gp120 vaccine boost adjuvanted with MF59 or alum in healthy adults without HIV (HVTN 107): A phase 1/2a randomized trial
Source: PLoS Med. 2024 Mar 19;21(3):e1004360. doi: 10.1371/journal.pmed.1004360 (PMC10986991; doi:10.1371/journal.pmed.1004360)

Figure S5. Comparison of serum V1V2 IgG binding antibody magnitude-breadth of geometric mean MFI (AUC). A. Month 6.5. B. Month 12. C. Month 12.5. D. Month 18. The V1V2 panel size of 6 includes the following antigens: gp70_B.CaseA_V1_V2, C.1086C_V1_V2 Tags, gp70_B.CaseA2 V1/V2/169K, gp70-TV1.GSKvacV1V2/293F, gp70-96ZM651.02 V1v2, AE.A244 V1V2 Tags/293F.

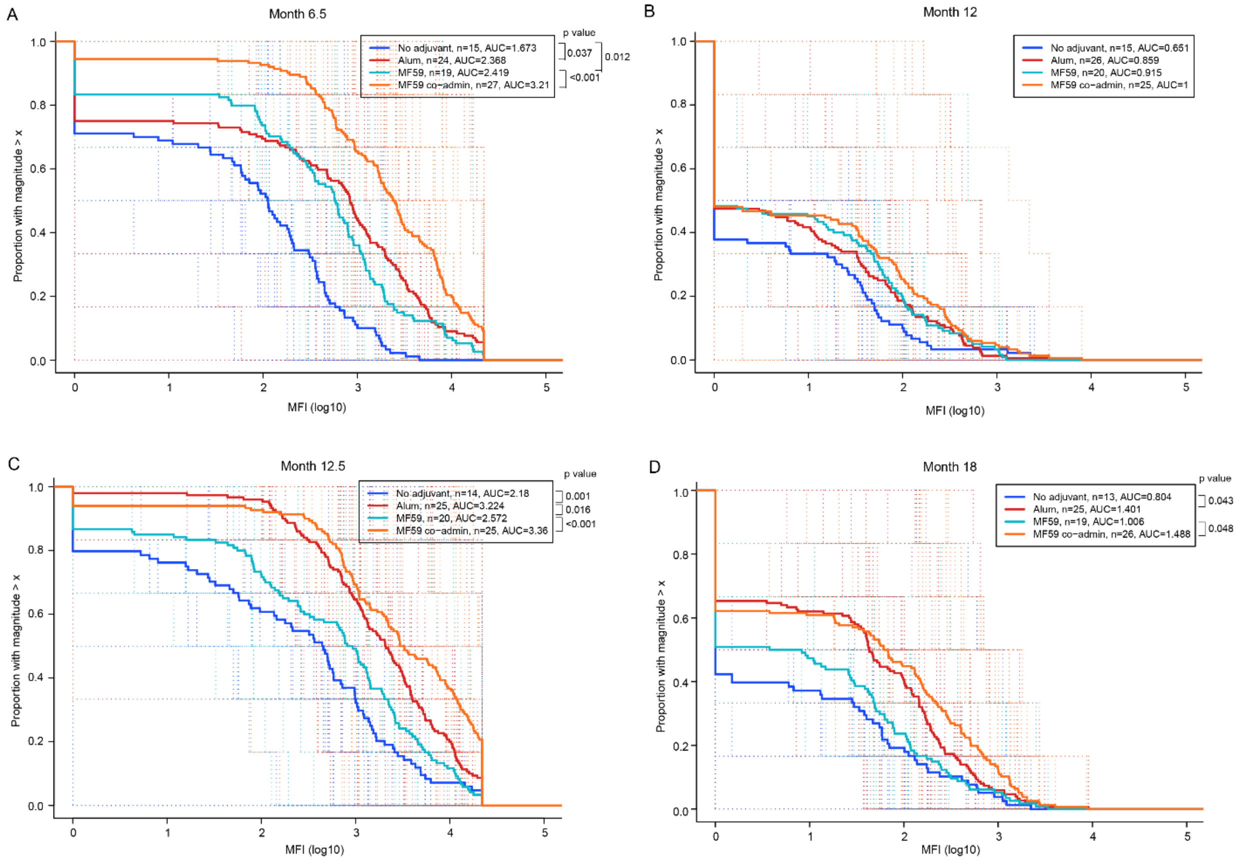

Supplement: S5 Fig — (PDF) [file pmed.1004360.s010.pdf]
