# Supplementary material for: Safety and immunogenicity of a subtype C ALVAC-HIV (vCP2438) vaccine prime plus bivalent subtype C gp120 vaccine boost adjuvanted with MF59 or alum in healthy adults without HIV (HVTN 107): A phase 1/2a randomized trial
Source: PLoS Med. 2024 Mar 19;21(3):e1004360. doi: 10.1371/journal.pmed.1004360 (PMC10986991; doi:10.1371/journal.pmed.1004360)

Figure S7. Response rates and boxplots of binding antibody IgG3 responses to the 3 gp120 vaccine-matched antigens at Months 6.5, 12, 12.5, 18. Significant p-values are shown for comparisons of response rates by Barnard's exact test and magnitudes among positive responders by Wilcoxon test for the following groups: MF59 vs. none, alum vs, none, MF59 vs. alum, MF59 vs. MF59 co-admin.

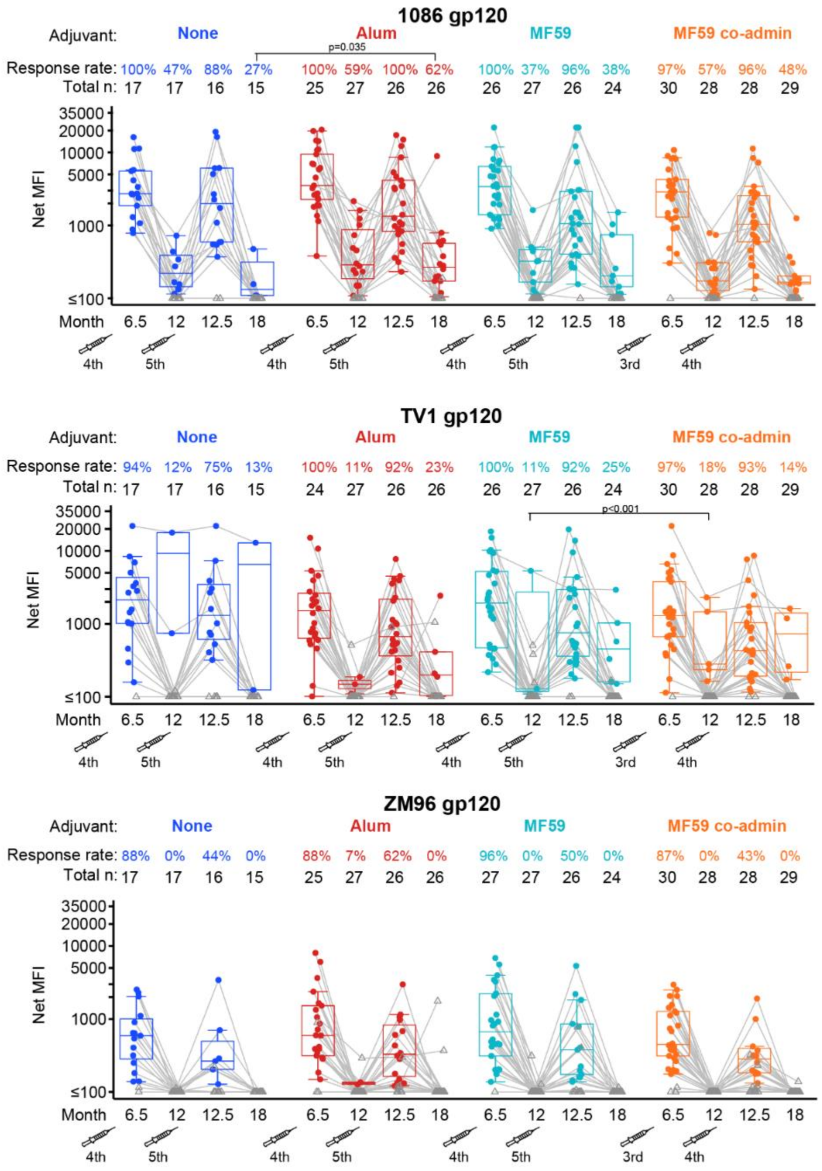

Supplement: S7 Fig — (PDF) [file pmed.1004360.s012.pdf]
