# Supplementary material for: Safety and immunogenicity of a subtype C ALVAC-HIV (vCP2438) vaccine prime plus bivalent subtype C gp120 vaccine boost adjuvanted with MF59 or alum in healthy adults without HIV (HVTN 107): A phase 1/2a randomized trial
Source: PLoS Med. 2024 Mar 19;21(3):e1004360. doi: 10.1371/journal.pmed.1004360 (PMC10986991; doi:10.1371/journal.pmed.1004360)

Figure S10. Heatmaps of median magnitude of CD4+ T-cell response by marker subset to vaccine-matched antigens at Months 6.5, 12, 12.5 and 18 for the marker subsets analyzed by COMPASS.

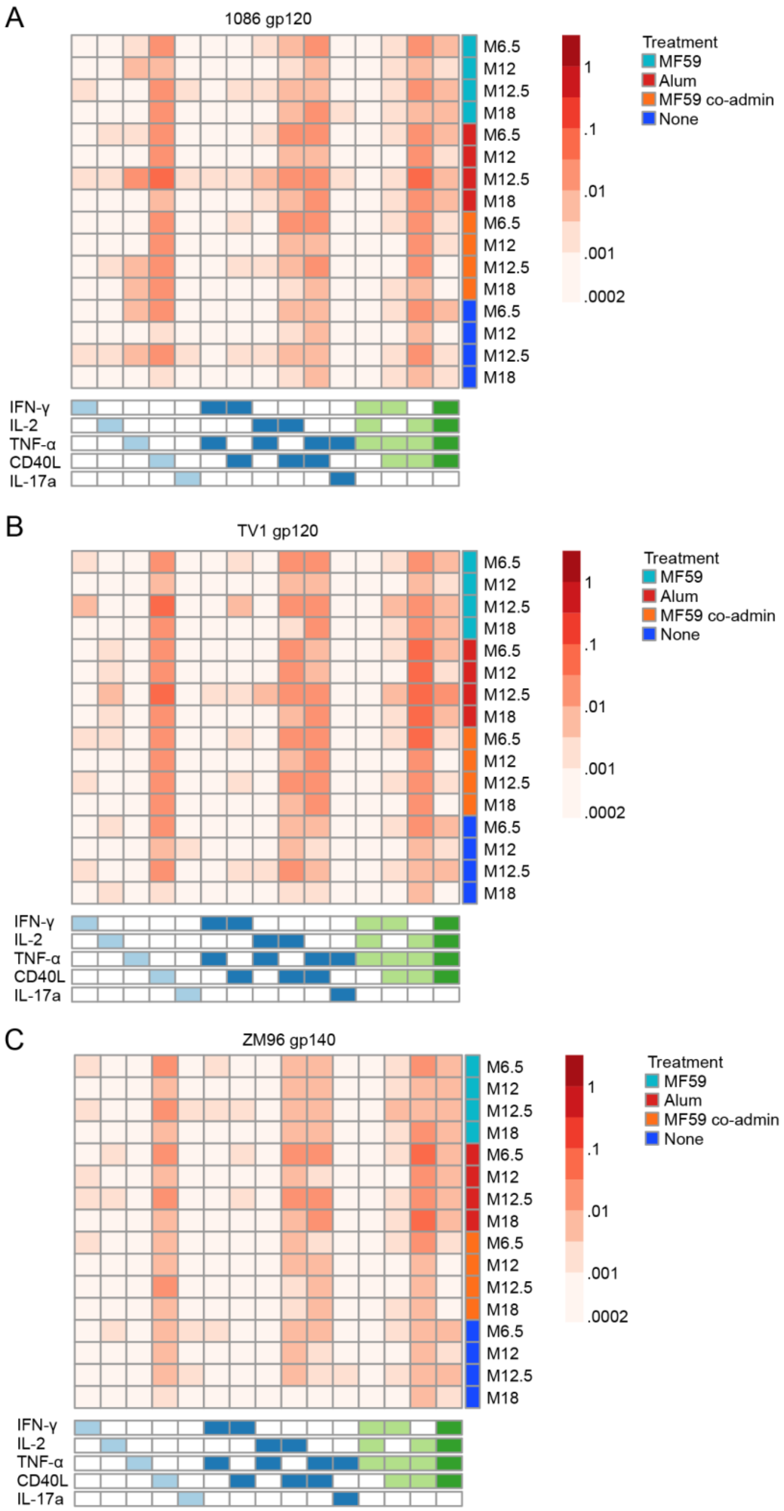

Supplement: S10 Fig — (PDF) [file pmed.1004360.s015.pdf]
