# Supplementary material for: Safety and immunogenicity of a subtype C ALVAC-HIV (vCP2438) vaccine prime plus bivalent subtype C gp120 vaccine boost adjuvanted with MF59 or alum in healthy adults without HIV (HVTN 107): A phase 1/2a randomized trial
Source: PLoS Med. 2024 Mar 19;21(3):e1004360. doi: 10.1371/journal.pmed.1004360 (PMC10986991; doi:10.1371/journal.pmed.1004360)
Supplement: Attachment — Submitted filename: PLOSMed_editorial_comments_letter_PMEDICINE-D-23-01761R1.docx [file pmed.1004360.s016.docx]

We thank the editorial board for their detailed request for additional information regarding the submission of our manuscript, **PMEDICINE-D-23-01761R1**. Please find our point-by-point responses to each comment in the italicized text below. Please also note that we have switched one of the co-authors: Dr. Nivashnee Naicker has replaced Dr. Nigel Garrett. Dr. Garrett recently brought to our attention that although he has served as eThekwini site principal investigator (PI) for many other HVTN trials, Dr. Naicker was the site PI for HVTN 107 and should be included in his place as co-author. All co-authors have approved this change.

---------------------------------------------------------------------------------------------

Editorial comments:

**Your study must strictly adhere to CONSORT reporting.**

*We have adhered to CONSORT reporting and include the completed CONSORT checklist in the Supplement as requested.*

**We request you to include a standalone 'Outcomes' section in the methods.**

*The following Outcomes section has been added to the manuscript:*

*“*The prespecified primary clinical objectives of the trial were to assess safety and tolerability of each vaccine regimen. Primary endpoints were local and systemic reactogenicity signs and symptoms, laboratory measures of safety, adverse events (AEs) and serious AEs (SAEs). The prespecified primary immunogenicity objectives were to compare the IgG bAb response rates and magnitudes among positive responders to 1086, TV1, and ZM96 gp120 of the ALVAC-HIV+gp120/MF59 vs. ALVAC-HIV+gp120/alum groups at month 6.5, and to compare IgA bAb response rates and magnitudes among positive responders to 1086, TV1, and ZM96 gp120 of the ALVAC-HIV+gp120/MF59 vs. ALVAC-HIV+gp120/MF59-co-administration groups at month 6.5. Secondary objectives were to evaluate the IgG and IgG3 bAb responses to Env gp120 and V1V2 antigens and CD4+ T cell responses to vaccine-matched antigens for each group at months 6.5, 12, 12.5, 18*.”*

**The Abstract should report only the primary outcome or must include ALL secondary outcomes (also see the statistical reviewer comments).**

*The primary outcomes of the trial were safety, IgG and IgA binding antibody responses to gp120 vaccine-matched antigens at Month 6.5. These are now noted and reported in the abstract.*

**In its current form, the trial is very difficult to follow and this needs to be resolved.**

*We have substantially revised the manuscript to help readers by streamlining the results in the Abstract and in the main text, adding a new Outcomes section to the Methods, a description of the comparisons of interest to the Statistical Analysis section of the Methods, and subheadings throughout the Results to note the outcomes described.*

**Please also indicate whether there were any deviations from the protocol.**

*There were no deviations from the prespecified primary and secondary objectives in the protocol.*

**Insert in the method section a paragraph to explain trial registration error and explain why only 3 months after patient participation the trial was registered on ClinicalTrials.gov.**

*A paragraph has been added to the Methods section:*

*“*HVTN 107 was prospectively registered with the South African National Clinical Trials Registry (SANCTR) on October 7, 2014 (see S4). The SANCTR registry date of October 14, 2019 is incorrect as the system would not have allowed an anticipated start date and last follow-up date to have occurred prior to the date of registration. This error likely occurred during SANCTR’s migration of all previously registered trials to a new platform. The protocol underwent two revisions prior to study start. Protocol version 3.0 was written on May 4, 2017, with approval received on June 13, 2017, before the first participant enrolled on June 19th, 2017 (see S1-S3 for original protocol, updated protocols and IRB approvals). The trial was also posted on the US registry, ClinicalTrials.gov on September 15, 2017. Prospective registration on ClinicalTrials.gov was not required given the trial was prospectively registered with SANCTR, was conducted entirely outside of the United States, and did not involve an Investigational New Drug (IND) submission to the US Food and Drug Administration (FDA) (see S5 for additional details)*.”*

*Additional information provided in the Supplement (S5 text):*

*“*The HVTN 107 trial was not prospectively registered on clinicaltrials.gov prior to enrollment of the first study participant due to the HVTN coordinating center, as the delegated responsible party, having made changes to its submission process that resulted in delays to completing the registry entry by study start. Unlike most other HVTN trials, this was a non-IND trial where the sponsor, NIAID DAIDS, was not responsible for trial registration in the US. Thus, there was a delay of about 12 weeks between the first participant enrolled on June 19, 2017 and the clinicaltrials.gov trial registration posting on September 15, 2017. However, the trial was prospectively registered with SANCTR well in advance of study start. None of the primary study outcomes were revealed during the short period of time in which the study was not registered on the US public clinical trial registry; enrollment was still ongoing with completion on June 14, 2018*.”*

**For all protocols, approvals, and registration docs - please add these as SI, in addition to the Consort checklist and the ICMJE declaration form (see below for more details)**

*The protocols, approvals, registration documents, Consort checklist and ICMJE declaration form have been added to the Supplement.*

**---------------------------------------------------------------------------------------------**

**GENERAL: Please provide 95% CIs and p values for all results were appropriate (including the abstract), check and amend throughout. We suggest reporting statistical information in the following format: ‘x’; (95% CI [‘y’,’ z’] p value) For p values, please report as p<0.001 and where higher as 'p=0.002'. Please add the statistical method used to your method section.**

*We have added 95%CIs to accompany p-values throughout where appropriate. P-values are reported in the requested format. All statistical methods that were used are described in the Statistical Analysis section of the Methods.*

**STUDY DESIGN:**

**Please complete the CONSORT checklist and ensure that all components of CONSORT are present in the manuscript, including how randomization was performed, allocation concealment, blinding of intervention, definition of lost to follow-up, power statement, etc.**

**When completing the checklist, please use section and paragraph numbers, rather than page numbers.**

*The CONSORT checklist has been completed, using section and paragraph numbers, and is included in the Supplement.*

**Please include all protocols and IRB approvals as SI.**

*These are included in the Supplement.*

**PLOS Medicine requires that all trials be prospectively registered in one of registries recognized by WHO. Please provide information on study registration in the Methods section. In your case, please insert a paragraph explaining the trial error date in SANCTR, and provide an explanation on why registration on clinical trials took place 3 months after participant recruitment had started, as to provide clarity to the reader. (As per previous communication and supplied information).**

*This has been added to the Methods and detailed in the Supplement.*

**We also request you to include a standalone 'Outcomes' section in the methods - in line with the study protocol. Please also highlight any deviations that may have occurred. Please also note if your trial had to undergo important modifications in response to extenuating circumstances, please complete the CONSERVE-CONSORT checklist and provide in your Supporting Information. As above, when completing the checklist, please use section and paragraph numbers, rather than page numbers.**

*An ’Outcomes’ section has been added to the Methods that is aligned with the study protocol’s primary and secondary objectives. There were no deviations from the prespecified primary and secondary objectives.*

*The CONSORT checklist has been completed, using section and paragraph numbers, and is included in the Supplement.*

**Furthermore, in accordance with ICMJE requirements, PLOS Medicine requires prospective, public registration of a data sharing plan (as part of mandatory clinical trials registration) for all clinical trials that began enrollment on or after January 1, 2019. Please add your plan as SI.**

*We have added our data sharing plan to the Supplement:*

**“*Data sharing***

*Data and protocols for HVTN 107 will be made publicly available online at* [*https://atlas.scharp.org/cpas/project/HVTN%20Public%20Data/begin.view*](https://atlas.scharp.org/cpas/project/HVTN%20Public%20Data/begin.view). “

**TITLE: Please revise your title according to PLOS Medicine's style. It should begin with main concept if possible and please place the study design ("A randomized controlled trial," "A retrospective study," "A modelling study," etc.) in the subtitle (ie, after a colon).**

*The title has been revised to:*

*“Safety and immune responses among healthy adults without HIV in HVTN 107: A phase 1/2a randomized trial of a subtype C ALVAC-HIV (vCP2438) and bivalent subtype C gp120 vaccines adjuvanted with MF59 or alum”*

**FINANCIAL STATEMENT/COMPETING INTERESTS:**

**I appreciate that some form of private funding is involved. I also note that ‘GlaxoSmithKline Biologicals SA was provided the opportunity to review a preliminary version of this manuscript for factual accuracy’ can you be more precise (e.g. who and level of changes made?) Would it be correct to state that ‘The funders had no role in the data collection and analysis, and the decision to submit for publication.’? If so, please include this line**

*We clarified that GlaxoSmithKline Biologicals SA co-authors were provided the opportunity to review the manuscript for factual accuracy and we also clarified the extent of their suggestions. We also noted that the funders had no role in data collection and analysis or the decision to submit for publication:*

*“*GlaxoSmithKline Biologicals SA co-authors were provided the opportunity to review this manuscript for factual accuracy and made minor edits to the product description and typographical edits, but the authors are solely responsible for final content and interpretation. The funders had no role in the data collection, analysis, and the decision to submit for publication.”

**We also invite you to complete the ICMJE (**[**https://www.icmje.org/disclosure-of-interest/**](https://urldefense.com/v3/__https:/www.icmje.org/disclosure-of-interest/__;!!GuAItXPztq0!gdiTyB-cddhKYbL_KJx3fxO0QHcJlHIY3FRgdEzU5LDWGfGRAJaJevvOeBVV6TJIi6s2ZC7UIjML$)**), which should include details of all authors’ interests, and please add your ICMJE statements as an SI**

**ETHICAL STATEMENT:**

**Please provide the name of each ethics board (and approval code if available) that approved the study.**

*We have added the names of each ethics board and number to the Ethics Statement:*

“*Initial and ongoing approvals of the study protocol and research review (see S1-S3) were provided by the Medicine Control Council (20141022), the Fred Hutchinson Cancer Research Center institutional review board (IRB), and local research ethics committees for each site: National Health Bioethics Committee (CNBS) (Ref 125/CNBS/2014), University of Cape Town Human Research Ethics Committee (Ref 790/2014), University of Witwatersrand Human Research Ethics Committee (Medical) (#141108), University of Kwazulu-Natal Biomedical Research Ethics Committee (Ref BFC453/14), University of Zimbabwe Joint Research Ethics Committee (Ref 186/15). All participants gave written informed consent in a language of their choice prior to implementation of study procedures.”*

**DATA AVAILABILITY:**

**"Manuscript data will be available upon request” is not an acceptable data availability declaration and will require revision.**

**For each data source used in your study:**

**a) If the data are freely or publicly available, note this and state the location of the data: within the paper, in Supporting Information files, or in a public repository (include the DOI or accession number).**

**b) If the data are owned by a third party but freely available upon request, please note this and state the owner of the data set and contact information for data requests (web or email address). Note that a study author cannot be the contact person for the data.**

**c) If the data are not freely available, please describe briefly the ethical, legal, or contractual restriction that prevents you from sharing it. Please also include an appropriate contact (web or email address) for inquiries (again, this cannot be a study author).**

**For more information, please consult** [**https://journals.plos.org/plosmedicine/s/data-availability#:~:text=PLOS%20journals%20require%20authors%20to,obtain%20access%20to%20the%20data**](https://urldefense.com/v3/__https:/journals.plos.org/plosmedicine/s/data-availability*:*:text=PLOS*20journals*20require*20authors*20to,obtain*20access*20to*20the*20data__;I34lJSUlJSUlJQ!!GuAItXPztq0!gdiTyB-cddhKYbL_KJx3fxO0QHcJlHIY3FRgdEzU5LDWGfGRAJaJevvOeBVV6TJIi6s2ZBYNBFQa$)**.**

*We have included the following data sharing statement in the Supplement:*

***“Data sharing***

*Data and protocols for HVTN 107 will be made publicly available online at* [*https://atlas.scharp.org/cpas/project/HVTN%20Public%20Data/begin.view*](https://atlas.scharp.org/cpas/project/HVTN%20Public%20Data/begin.view)*”*

**ABSTRACT:
Please report your abstract according to CONSORT for abstracts** [**https://www.equator-network.org/reporting-guidelines/consort-abstracts/**](https://urldefense.com/v3/__https:/www.equator-network.org/reporting-guidelines/consort-abstracts/__;!!GuAItXPztq0!gdiTyB-cddhKYbL_KJx3fxO0QHcJlHIY3FRgdEzU5LDWGfGRAJaJevvOeBVV6TJIi6s2ZObYd-9T$) **and the abstract should report only the primary outcome or must include ALL secondary outcomes**

**Please also structure your abstract using the PLOS Medicine headings: Background, Methods and Findings, Conclusions. Please remove all other subheaders.

Abstract Background:
Provide the context of why the study is important. The final sentence should clearly state the study question.**

*This has been done.* **Abstract Methods and Findings:
Please include the study design, population and setting, number of participants, years during which the study took place, length of follow up, and main outcome measures.
Please quantify the main results (with 95% CIs and p values).
In the last sentence of the Abstract Methods and Findings section, please describe the main limitation(s) of the study's methodology.**

*These changes have been made.* **Abstract Conclusions:
Please address the study implications without overreaching what can be concluded from the data.
Please interpret the study based on the results presented in the abstract, emphasizing what is new without overstating your conclusions.

Please include the clinical trial registry number at the end of the abstract.**

*These changes have been made.*

**AUTHORS SUMMARY:**

**We also ask that you include a short, non-technical Author Summary of your research to make findings accessible to a wide audience that includes both scientists and non-scientists. The Author Summary should immediately follow the Abstract in your revised manuscript. This text is subject to editorial change and should be distinct from the scientific abstract. Please see our author guidelines for more information:** [**https://journals.plos.org/plosmedicine/s/revising-your-manuscript#loc-author-summary**](https://urldefense.com/v3/__https:/journals.plos.org/plosmedicine/s/revising-your-manuscript*loc-author-summary__;Iw!!GuAItXPztq0!gdiTyB-cddhKYbL_KJx3fxO0QHcJlHIY3FRgdEzU5LDWGfGRAJaJevvOeBVV6TJIi6s2ZDl0RYTG$)

**Ideally each sub-heading should contain 2-3 single sentence, concise bullet points containing the most salient points from your study.**

**In the final bullet point of ‘What Do These Findings Mean?’ Please include the main limitations of the study in non-technical language.**

*The Author Summary has been added, following the above guidelines.*

**DISCUSSION:**

**Please present the Discussion as follows: a short, clear summary of the article's findings; what the study adds to existing research and where and why the results may differ from previous research; strengths and limitations of the study; implications and next steps for research, clinical practice, and/or public policy; followed by a one-paragraph conclusion.**

*These guidelines have been followed.*

**ACKNOWLEDGMENTS/ DECLARATIONS**

**Please remove all statements apart from acknowledgements, author contributions and abbreviations from the end of the main manuscript and include these only in the relevant parts of the manuscript submission form. Funding, competing interest, and data availability will be compiled as metadata.**

*These guidelines have been followed.*

**REFERENCES:**

**Please use the "Vancouver" style for reference formatting, and see our website for other reference guidelines** [**https://journals.plos.org/plosmedicine/s/submission-guidelines#loc-references**](https://urldefense.com/v3/__https:/journals.plos.org/plosmedicine/s/submission-guidelines*loc-references__;Iw!!GuAItXPztq0!gdiTyB-cddhKYbL_KJx3fxO0QHcJlHIY3FRgdEzU5LDWGfGRAJaJevvOeBVV6TJIi6s2ZC30nuWf$)

**For in-text reference, citations are placed within square parentheses and should precede punctuation. Please amend throughout.**

*We have changed the formatting to reflect the Vancouver style for PLoS Medicine.*
