# Supplementary material for: Safety and immunogenicity of a subtype C ALVAC-HIV (vCP2438) vaccine prime plus bivalent subtype C gp120 vaccine boost adjuvanted with MF59 or alum in healthy adults without HIV (HVTN 107): A phase 1/2a randomized trial
Source: PLoS Med. 2024 Mar 19;21(3):e1004360. doi: 10.1371/journal.pmed.1004360 (PMC10986991; doi:10.1371/journal.pmed.1004360)
Supplement: Attachment — Submitted filename: PMEDICINE-D-23-03685R1_reviewer_response.docx [file pmed.1004360.s018.docx]

We thank the editors and reviewers for taking the time to provide final comments on our resubmitted manuscript, **PMEDICINE-D-23-01761R1**.

Please find our point-by-point responses to each comment in the italicized text below.

**Reviewer #1: Statistical review

This is a paper reporting a phase I/II trial comparing four different formulations of HIV-1 vaccine in terms of safety and vaccine response. I had reviewed the previous submission reporting this trial. Generally I found this resubmission easier to follow and my previous comments were addressed well.. The issues with registration of the trial raised also appear to be addressed.

I have a few additional minor comments on this submission.

1.      Introduction, line 155 "waning to 31.2% (95% CI: 1.1, 52.1, p-value = 0.04)" - I would remove the p-value here (or add the p-value to the previous line's result) to keep consistent.**

*Thank you, we have removed the p-value for consistency with the previous sentence.*

**2.      Methods line 247: it would be useful to provide some intuition as to why the ratio was 3:3:3:1 for individuals not in the additional sample subset.**

*We have added the following text to explain the ratio for the additional sample subset:*

*“Participants not in the additional sample subset (n=60) were randomized in a 3:3:3:1 ratio to the four vaccine groups as the adjuvanted groups were of primary interest.”*

**3.      Line 328-329: personally I would recommend the sample size calculation is featured in the main paper.**

*Thank you, we have added the sample size calculations to the Statistical Analysis section in the main paper.*

**4.      Line 475 - I think there's a typo here with the median difference and 95% CI for month 18.**

*Thank you for catching this typo. The 95% CI lower bound has been corrected.*

**5.      Line 495: I do not think PLOS medicine permits 'data not shown'.**

*We have added a Table S4 of CD8+ responses to TV1 gp120 to support the statement.*

**James Wason


Reviewer #2: Dear authors and editor:

All raised comments have been addressed and implemented properly in the revised version of the manuscript. Results of the study are now better presented, more structured and the manuscript has overall improved in its clarity for the reader. The work address a very critical question in the field for vaccine regimen design; both in terms of adjuvant selection and prime/boost timings. Although sample size is an important limitation of the trial, it is unlikely that a bigger trial would be ever be designed and would provide different evidence that the current trial. Overall I think it's worth its publication. Thanks for the opportunity to review this work.**

*Thanks very much for your comments.*

**Reviewer #3: The MS is very much improved. How ever one minor point is that the antigenic contents in GP 120 C plus MF 59 is only 100 mcg. the gp 120 B/E plus Alum used in RV 144 was 300 mcg.
Not sure whether authors would like to add discussion on this.**

**------------------------------------------------------------
Requests from Editors:

Editorial requests:

1. Title: We suggest that the title is modified slightly to better conform to PLOS Medicine style. Eg, “Safety and immunogenicity of a subtype C ALVAC-HIV vaccine prime plus bivalent subtype C gp120 vaccine boost adjuvanted with MF59 or alum in health adults without HIV (HVTN 107): A phase 1/2a 2 randomized trial” (or similar).**

*Thank you, we have changed the title as suggested to: Safety and immunogenicity of a subtype C ALVAC-HIV (vCP2438) vaccine prime plus bivalent subtype C gp120 vaccine boost adjuvanted with MF59 or alum in healthy adults without HIV (HVTN 107): A phase 1/2a randomized trial.*

**2. Abstract/background: Regarding this text, “Our trial directly compared immune responses elicited by alum vs. MF59 adjuvants in the RV144-like HIV vaccine regimen modified for the Southern African region; RV144 is the only trial to have shown modest HIV vaccine efficacy. Data generated after RV144 suggested that use of MF59 adjuvant might allow lower protein doses to be used.”

a. The composition of the RV144 vaccine needs to be introduced briefly in the abstract (as you have done in the introduction);**

*This has been added to the background of the abstract.*

**b. Please indicate whether the RV144 vaccine regimen was adjuvanted with alum, as is implied in the Introduction, lines 160-161 (“…replaced the alum adjuvant with MF59…”);**

*This has been added to the background of the abstract.*

**c. Please add an additional sentence to explain the change of adjuvant in the new regimen with regard to the expectation that MF59 would enhance immunogenicity and why (ie, results from NHP studies). This addition is also needed to put into context the first sentence of the Abstract conclusion (otherwise this is the first mention of the expectation that MF59 would enhance responses).**

*We have now included the sentence: “Data generated from non-human primate studies after RV144 suggested that use of MF59 adjuvant might allow lower protein doses to be used while maintaining robust immune responses.”*
 **3. Abstract/background, lines 90-91: Please delete “with one less dose” for simplicity; this detail is made clear later and isn’t needed to understand the trial design;**

*This edit has been made.*

**4. Abstract, lines 102-103: Please slightly revise to “Primary outcomes were safety and occurrence and mean fluorescence intensity (MFI) of…”**

*This edit has been made.*

**5. Author summary/What did the researchers do and find: “100% of all vaccinees” should be revised to “All vaccines” or “100% of vaccines” (ie, “all” and “100%” are redundant).**

*This edit has been made.*

**6. Introduction, first sentence: Please briefly introduce the RV144 vaccine (vaccine components and regimen) for those who are not familiar with the details of these trials (eg, ALVAC-HIV at mo 0 and 1, ALVAC-HIV + bivalent gp120 boost at mo 3 and 6).**

*This text has been added.*
 **7. Introduction, line 165: I would slightly rephrase this sentence for clarity; eg, “ Differences between the vaccine efficacy and immune correlates of HIV-1 acquisition risk reported in the RV144 trial in Thailand and the HVTN 702 trial in South Africa (and other similar pox-protein HIV-1 vaccine regiments) have left open questions for the HIV vaccine field.”**

*This edit has been made.*  **8. Introduction, line 172: Please add references 13-16 at the end of this statement for clarity: “In response to findings from nonhuman primate (NHP) studies…”**

*This edit has been made.* **9. Introduction, lines 177-178: “However, use of the original alum adjuvant conferred protection from low dose SIVmac251 or SHIV(AD8) challenge.” Did the RV144 regimen adjuvanted with MF59 *not* confer protection in NHP, or was this not tested?**

*We have clarified that protection was not seen with MF59, only with the alum-adjuvanted regimen.*
 **10. Introduction, line 182: “…the MF59 vs. alum adjuvant question was addressed in the parallel HVTN 107 trial.” By ‘parallel’ do you mean parallel to HVTN 702? It would be useful if this was made more explicit that these two trials ran in parallel.**

*Thank you, the timing of the two trials has now been clarified in the text.*

**11. Introduction, lines 185-188: This sentence is confusing as written; I suggest dividing it into 2 sentences. Eg, “Subsequent analyses of blood samples from participants in the RV144 and HVTN 702 trials highlighted the important role of IgG binding antibodies specific for Env variable regions 1 and 2 (V1V2) and vaccine-specific CD4+ T-cell responses in protection from HIV-1 acquisition [21,22]. Here we report here on the safety and immunogenicity of the HVTN 107 vaccine regimen in view of those data on correlates of protection.”**

*This edit has been made.*

**12. Methods, line 239: Please clarify the following sentence: “During the consent process, participants could choose whether to enrol in a subset to provide additional samples.” I would suggest something like, “During the consent process, participants could choose whether to enrol in a study subset to provide additional samples for the assessment of innate and mucosal immune responses.”**

*This edit has been made.*

**13. Method (general): please add sample size calculation, demonstrating that the trial was adequately powered. Please move relevant text from supplement to main text.**

*The relevant text has been moved to the Statistical Analysis section.*

**14. Results, line 347. Please move Table S2 to the main body of the manuscript. The baseline characteristics table should be presented in the main paper, per CONSORT.**

*This change has been made; it now appears as Table 1.*

**15. Results, overall primary and secondary outcomes sub-headers comment: I wonder if it may be better to have one single ‘Primary outcomes’ and one single ‘Secondary outcomes’ sub-header without the additional information being part of the sub-headers, as this feels not needed (and makes for heavy reading). Please remove all other sub-headers in the Immunogenicity section (apart from one single ‘Primary outcomes’ and one single ‘Secondary outcomes’).**

*These edits have been made.*

**16. Results, lines 388-390: The following sentence should be moved to the introduction: “HIV-1–specific IgG and IgG3 serum bAb responses to HIV-1 envelope antigens correlated with reduced HIV-1 acquisition in the RV144 trial [21, 28, 32] and were therefore of interest in our study.” In addition, the wording makes it sound as if this were tested post-hoc, but these endpoints were pre-specified, so you might wish to rephrase this.**

*This sentence has been moved to the Introduction and revised.*

**17. Results, line 393-395, please include a call-out to the appropriate figure referenced in this statement: “Overall, gp120-directed responses to these antigens were greater than the V1V2-directed responses, with considerable waning of all responses 6 months following the month 12 vaccination.”**

*A call-out to the appropriate figures has been added.*

**18. Results, line 454. This sentence starts with “Finally, we compared …” yet in the next paragraph you compare T-cell responses in ALVAC-HIV+gp120/MF59 or ALVAC482 HIV+gp120/alum vs. ALVAC-HIV+gp120/no-adjuvant groups. Please amend or make clear by the first 3 comparatives are more closely related.**

*Thank you. We have revised this to “Next, we compared…”* **19. Results, line 495. Data not shown is not permitted. Please add data to support this statement, or remove the statement.**

*We have added data to support this statement in Table S4.*

**20. Discussion, lines 535-536: The following sentence should be removed from the discussion: “The publication of NHP studies that assessed the effect of alum and MF59 adjuvants on immunogenicity and SHIV/SIV acquisition motivated the HVTN 107 study”. This fits better in the introduction, where it is more elaborated upon. You can start this section with “ This study sought to determine … .” (line 536).**

*This sentence has been removed from the Discussion. It was not added to the Introduction, as the point is already covered.*

**21. Discussion, line 553. “. Unlike the NHP studies’ findings, …” Please insert reference(s) for the NHP studies.**

*References have been added.*

**22. Figures. There are a large number of figures currently included in the main paper. Please consider whether any of the figures could be moved to the supplement.**

*We have moved two of the figures to the Supplement.* **23. Please ensure you have redefined abbreviations in the captions of your figures and legends (e.g see S1 Table).**

*We have made these edits.*

**24. Please remove the financial statement from the end of the manuscript and include this only in the relevant parts of the manuscript submission form. The financial statement will be compiled as metadata.**

*We have made this edit.*

**25. Please remove page numbers from the CONSORT checklist (your published article will not contain page numbers)**

*Page numbers have been removed from the CONSORT checklist.*
